# New to Science: Insect Discoveries from Long-Term Monitoring in Terrestrial Protected Areas in Abu Dhabi, UAE

**DOI:** 10.3390/insects17101083

**Published:** 2026-10-19

**Authors:** Anitha Saji, Zamzam Salem Al Rashdi, Shakeel Ahmed, Ahmed Ali Mohamed, Shaikha Salem Al Dhaheri

**Affiliations:** Terrestrial and Marine Biodiversity Sector, Animal Assessment and Monitoring, Environment Agency-Abu Dhabi, Abu Dhabi P.O. Box 45553, United Arab Emirates; zamzam.alrashdi@ead.gov.ae (Z.S.A.R.); akhan@ead.gov.ae (S.A.); ahmed.mohamed@ead.gov.ae (A.A.M.); saldhaheri@ead.gov.ae (S.S.A.D.)

**Keywords:** insect biodiversity, new species, new genus, taxonomy, Hymenoptera, Diptera, Neuroptera, malaise traps, protected areas, long-term monitoring, Abu Dhabi, United Arab Emirates

## Abstract

In 2005, the known insect fauna of the United Arab Emirates comprised approximately 800 species. Through the Invertebrate Inventory Project, initiated by the Environment Agency–Abu Dhabi (EAD), a comprehensive inventory of insect diversity within the Emirate’s protected areas has been conducted for more than two decades. Among the taxa documented through these surveys are 26 insect species and one genus, comprising 22 wasp species (Hymenoptera), three species of true flies (Diptera), and one antlion species (Neuroptera). Of these 26 species, 25 have been formally described in previous taxonomic publications based on material collected through the EAD inventory, whereas one Drapetis taxon from Al Wathba Wetland Reserve remains unpublished and is not treated here as an available nomenclatural act under the International Code of Zoological Nomenclature (ICZN). The genus and its associated species included in the synthesis were originally established in previous taxonomic publications. Malaise traps were used as the primary sampling method, complemented by pitfall traps, water traps, and other standard entomological trapping techniques. This study consolidates the available taxonomic information, distributional records, ecological observations, and biogeographical insights for these taxa. The long-term inventory highlights the effectiveness of systematic sampling for documenting previously unrecognized insect diversity and emphasizes the importance of terrestrial protected areas in revealing and conserving the largely understudied invertebrate biodiversity of the United Arab Emirates.

## Simple Summary

Abu Dhabi’s protected areas contain a rich but still poorly understood diversity of insects. For more than 20 years, the Environment Agency–Abu Dhabi has been carrying out a long-term survey to document insect species across these important habitats. This study reports 26 insect species previously unknown to science, including one species belonging to a newly described genus. The 26 species comprise 22 wasps, three flies, and one antlion. Most of these discoveries were made within protected areas, demonstrating their importance in safeguarding unique and endemic wildlife. The findings show that even in arid desert environments, many species remain undiscovered and that long-term monitoring is essential for understanding biodiversity. The study also highlights the scientific value of the United Arab Emirates, where species from different regions of Africa, Europe, and Asia meet and evolve. Insects play vital roles in pollination, nutrient recycling, and pest control, and maintain healthy ecosystems. As insect populations are declining worldwide because of habitat loss, climate change, and other human pressures, documenting and protecting species has become increasingly important. The results support continued biodiversity surveys, strengthened conservation measures, and highlight the importance of the Environment Agency-Abu Dhabi’s (EAD) ongoing efforts to document, discover, and maintain comprehensive biodiversity information, which is essential for evidence-based conservation and sustainable environmental management in the UAE.

## 1. Introduction

Insects are the most diverse group of animals on Earth and play indispensable roles in ecosystem functioning, contributing to essential processes such as pollination, nutrient cycling, decomposition, soil formation, and food-web dynamics. They are also widely used as indicators of environmental change because their abundance, diversity, and distribution can respond rapidly to changes in habitat conditions [1,2,3,4]. Despite their ecological importance, the insect fauna of arid and semi-arid regions remains relatively underexplored, particularly in the Arabian Peninsula. The United Arab Emirates (UAE), situated at the crossroads of the Palearctic and Afrotropical ecozones, encompasses a mosaic of habitats ranging from coastal areas and wetlands to desert plains, agricultural landscapes, and rugged mountains [5,6]. This environmental heterogeneity provides a range of habitats for a diverse insect fauna. Although the complete insect diversity of the UAE remains incompletely documented, at least 2588 insect species representing 23 orders have been recorded from the country [7]. Species-rich orders include Hymenoptera, Coleoptera, Diptera, and Lepidoptera, while other orders, including Orthoptera, Hemiptera, Neuroptera, Odonata, Mantodea, and Thysanoptera, are also represented. Several less extensively studied groups, including Collembola, Psocoptera, Ephemeroptera, Dermaptera, Phasmida, Embioptera, Isoptera, Phthiraptera, Strepsiptera, and Thysanura, further contribute to the overall entomofauna of the UAE [7]. Despite these records, substantial gaps remain in the taxonomic knowledge of the UAE insect fauna, particularly in protected areas and less intensively studied habitats. To address this need, the Environment Agency–Abu Dhabi (EAD) initiated a long-term insect inventory programme aimed at documenting and cataloguing insect diversity within the protected areas of the Abu Dhabi Emirate. The programme has involved sustained entomological surveys using a range of sampling approaches, including Malaise traps, light traps, pitfall traps, water traps, and targeted seasonal sampling. Such long-term surveys provide opportunities to detect previously undocumented taxa and improve knowledge of insect distributions in arid environments. One of the important sites included in this programme is Al Wathba Wetland Reserve (AWWR) (1), which is internationally recognized for its ecological importance [8]. The reserve contains a mosaic of terrestrial and aquatic habitats that supports a diverse insect assemblage. Surveys conducted across AWWR (1) and other protected areas of Abu Dhabi emirate have generated substantial material for taxonomic investigation and have contributed to the discovery of previously undocumented insect taxa.

This study synthesizes the results of extensive, long-term entomological surveys conducted within the protected areas of the Abu Dhabi Emirate. The collected material has contributed to the formal description of 26 new insect species in previous taxonomic publications, including one species belonging to a newly described genus. In addition, updated distributional information is provided for several previously known taxa. These findings demonstrate the importance of sustained insect surveys and taxonomic research for documenting insect diversity in arid ecosystems and highlight the value of protected areas in conserving and generating knowledge of the region’s insect fauna.

The Sheikh Zayed Protected Areas Network is a system of ecologically important conservation sites across Abu Dhabi, established and managed by the Environment Agency–Abu Dhabi [9]. These areas are critical for the protection of endangered and endemic species, such as the Arabian oryx, Arabian sand cat, houbara bustard, and numerous invertebrates and plant species (Figure 1). This network also serves as a foundation for long-term biodiversity monitoring programs, including studies on insects and other invertebrates, contributing to new species discoveries and evidence-based conservation planning in the UAE. Insect material examined in this study was collected from eight terrestrial protected areas and one non-protected area across the Emirate of Abu Dhabi, United Arab Emirates, during the period of 2002–2023. The specimens were obtained as part of long-term biodiversity surveys conducted by the Terrestrial and Marine Biodiversity Sector of EAD under the invertebrate inventory project. These surveys aimed to document the diversity and distribution of terrestrial arthropods within protected areas, with a focus on native species and natural communities.

## 2. Materials and Methods

### 2.1. Study Area

This study considered the 16 protected areas in the Emirate of Abu Dhabi, UAE with insect sampling conducted in eight of these protected areas and at one additional non-protected site, Al Dhafra Region. The study aimed to document insect diversity and examined the geographic distribution of insect species described as new to science based on specimens collected within the emirate (Figure 1). The 16 protected areas considered were (1) Al Wathba Wetland Reserve, (2) Al Houbara Protected Area, (3) Al Beda’a Protected Area, (4) Barqa Al Soqour Protected Area, (5) Al Tawi Protected Area, (6) Arabian Oryx Protected Area, (7) Qasr Al Sarab Protected Area, (8) Jabal Hafit National Park, (9) Yaw Al Debsa Protected Area, (10) Al Ghada Protected Area, (11) Al Ramlah Protected Area, (12) Al Wathba Fossil Dunes, (13) Bida’ Hazza’ Protected Area, (14) Natural Ghaf Protected Area, (15) Artificial Recharge Protected Area, and (16) Al Delfaowyah Protected Are. Collectively, these study areas represent a range of environmental conditions and habitat types within the Emirate of Abu Dhabi. Several of the study areas are located within the Sheikh Zayed Protected Areas Network, a network of designated conservation sites established and managed by the Environment Agency–Abu Dhabi (EAD). Al Dhafra Region, however, is located outside the officially designated protected-area network. Therefore, the term study area is used throughout this manuscript to refer collectively to all nine localities, without implying that each locality is a formally designated protected area. The distribution of the study areas and associated collection localities is presented in (Figure 1) illustrating the geographic scope of sampling and the localities from which specimens contributing to the taxonomic description of insect species new to science were obtained.

### 2.2. Data Collection

The primary collection method employed was Malaise traps [10] deployed across diverse habitat types within the protected areas (Figure 2). Notably intensive sampling efforts were conducted in AWWR (1) and the Al Houbara Protected Area (2). The Malaise trap (Watkins & Doncaster, Leominster, United Kingdom) is a passive flight interception device particularly effective for collecting flying insects such as Diptera, Hymenoptera, and Hemiptera. The design exploits the phototactic behavior of insects: upon encountering the vertical black mesh, insects tend to fly upwards toward the white roof and are guided into a collection bottle positioned at the apex. The collection bottles were filled with 95% ethanol (Carlo Erba Reagents SAS, Cornaredo, Milan, Italy) which served as a preservative for both morphological and molecular studies. Traps were maintained continuously with collection bottles replaced biweekly to ensure sample integrity. All specimens were initially preserved in ethanol and later curated for taxonomic examination. Type specimens of all new species described herein are deposited in the Invertebrate Collection of the Environment Agency–Abu Dhabi (EAD-INV). Each specimen is assigned a unique collection number with the prefix “EAD-INV”. The collection is housed in the Head Quarter building, Terrestrial and Marine Biodiversity Sector (TMBS), Environment Agency—Abu Dhabi. Specimens were preserved in ethanol and processed following standard entomological protocols [11]. The specimens examined in this study were collected through long-term insect monitoring conducted by the EAD. Following completion of the respective taxonomic descriptions, the relevant EAD specimens, including paratypes, were returned to and are maintained in the Invertebrate Collection of the Environment Agency–Abu Dhabi (EAD-INV) as part of the EAD reference collection. No new species are formally described in the present work.

Specimen imaging was performed using a Leica DFC 420 digital camera mounted on a Leica M80 stereomicroscope (Leica Microsystems (Switzerland) Ltd., Heerbrugg, Switzerland). High-resolution images were generated using Combine ZP image-stacking software (version 1.0; Alan Hadley) to produce fully focused photographs suitable for detailed morphological examination. Species identifications and photographic documentation were undertaken by specialist entomologists with expertise in the insect fauna of the Arabian Peninsula.

Locality information was obtained from original specimen labels and cross-verified against the official toponyms of Abu Dhabi’s protected areas in the Emirate of Abu Dhabi [9] to ensure geographic accuracy. These verified records were used to generate distribution maps and assess biogeographic patterns across the surveyed protected areas. Additional ecological information for each sampling area was obtained from the corresponding protected area management plans and environmental reports. A representative image of the AWWR (1) (Figure 3) is presented because 15 of the 26 species formally described from specimens collected during the monitoring programme originated from this site, representing the highest number among the sampling sites.

## 3. Results

The invertebrate surveys, including Malaise trapping, were conducted between 2002 and 2023 across eight of the 16 declared protected areas in the Emirate of Abu Dhabi, together with one non-protected desert site (Figure 1).The surveys generated a substantial body of insect material that was subsequently examined through taxonomic studies and contributed to the description of multiple taxa from the emirate. The survey material comprised 26 insect species representing 13 families and three orders: 22 species of wasps (Hymenoptera), three species of true flies (Diptera), and one species of antlion (Neuroptera). Among these taxa, 25 species have been formally described in previous taxonomic publications based on material collected through the EAD Invertebrate Inventory Project.

The material also includes one Drapetis taxon collected from Abu Dhabi that remains unpublished and has not yet been formally described. This unpublished taxon is included here as inventory material and is not treated as an available nomenclatural act under the International Code of Zoological Nomenclature (ICZN). The formally described taxa were recorded across nine sampling localities, encompassing both protected and non-protected areas. AWWR recorded the highest number of species (15), followed by Al Houbara Protected Area and Al Bida’a Protected Area, with seven species each, and Barqa Al Suqoor Protected Area, with five species. Al Tawi Protected Area and Arabian Oryx Protected Area each recorded two species, whereas Al Dhafra Region of Abu Dhabi, Jabal Hafit National Park, and Yaw Al Dibsa Protected Area each recorded one species. Because some species were recorded from more than one locality, site-level species counts are not mutually exclusive and therefore do not sum to the total number of species documented in the study. Malaise traps were the principal sampling method contributing material to the taxonomic records, with pitfall and water traps providing additional specimens. The specimens generated through these surveys subsequently formed part of the taxonomic material used in the formal descriptions of the taxa included in this synthesis and are represented in the EAD Invertebrate Collection. Among the taxa resulting from the EAD Invertebrate Inventory Project is Atropatopsis Sudheer & Narendran [12] a genus established from specimens collected in Abu Dhabi, with *Atropatopsis abudhabiensis* Sudheer & Narendran [12] designated as its type species. The establishment of this genus represents one of the taxonomic outcomes documented from the material generated through the long-term inventory programme.

The present study consolidates taxonomic information dispersed across previous publications and provides a single account of the insect taxa described from specimens collected through the long-term EAD Invertebrate Inventory Project. The synthesis brings together information on the taxonomic affiliations, collection localities, protected area associations, sampling methods, and representation of the specimens in the EAD Invertebrate Collection. The original descriptions and authorship of each taxon are cited in the corresponding species accounts and reference list, while updated taxonomic, distributional, ecological, and biogeographical information is synthesized here. Photographic documentation of the taxa is provided in Figure 4, Figure 5 and Figure 6.

No new taxa or nomenclatural acts under the ICZN are proposed in the present study. The Drapetis taxon mentioned above remains unpublished and is therefore not treated as a formally described species. The results presented here should accordingly be considered a retrospective synthesis of previously published taxonomic records and associated inventory material generated through long-term insect monitoring in the Emirate of Abu Dhabi.

**Figure 4:**
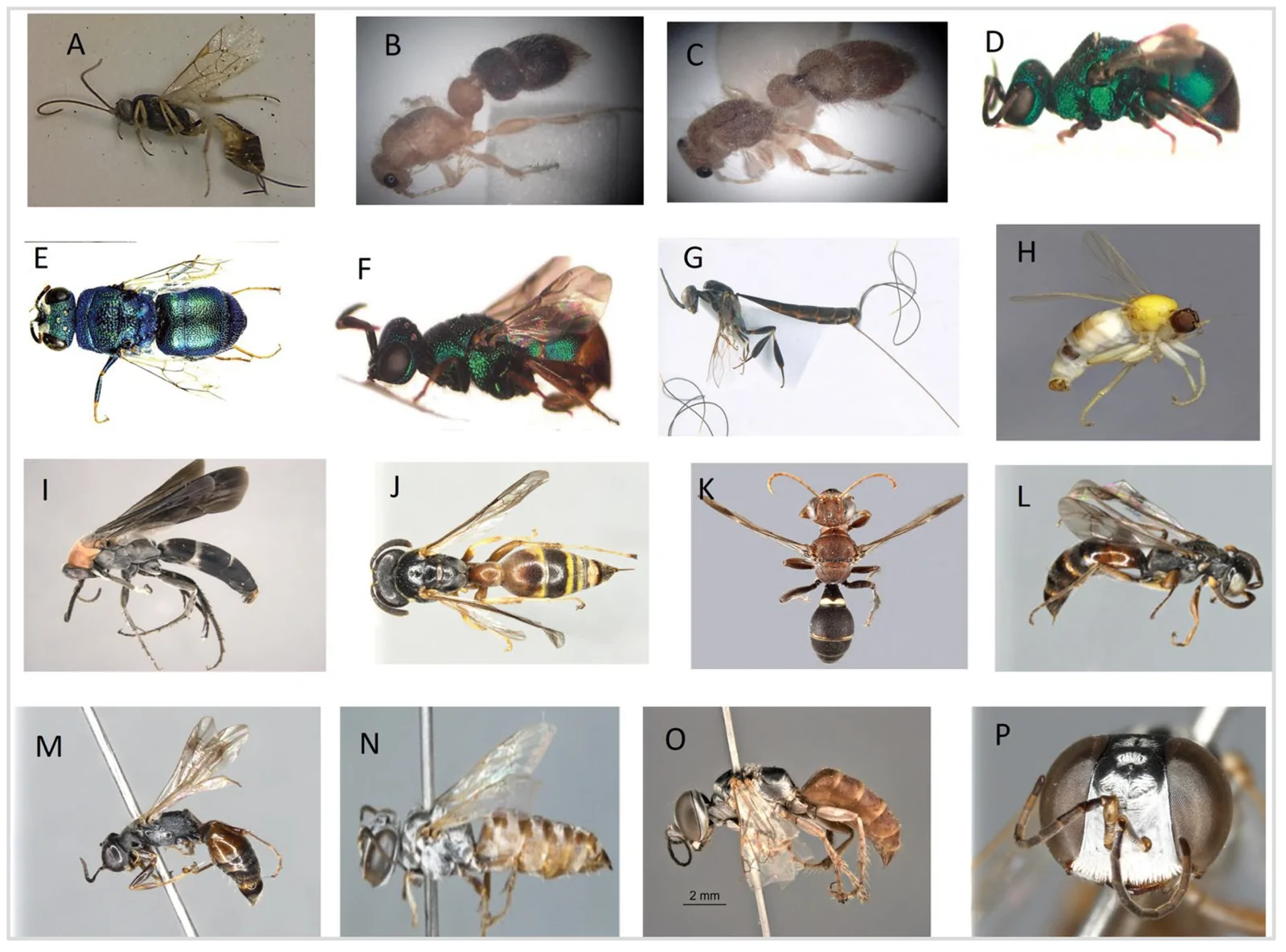
(**A**–**P**). *Atropatopsis abudhabiensis*; ♀, lateral view (**A**). *Macroocula tigarae*; ♀ holotype (**B**). *Macroocula sajia*; ♀ holotype (**C**). *Hedychridium anithaae*; ♀ holotype (**D**). *Chrysis houbaraeensis*; ♀ holotype (**E**). *Hedychridium caerulescens*; ♀ holotype (**F**). *Gasteruption alwathbaense*; ♀ holotype (**G**). *Drapetis wathabiensis*; ♀ holotype (**H**). *Ferreola alraeesii*; ♀ holotype (**I**). *Ammatomus wathabensis*; ♀ holotype (**J**). *Oryttus konradschmidti*; ♀ holotype (**K**). *Didineis mokrousovi*; ♀ holotype (**L**). *Gastrosericus albidaaensis*; ♂ holotype (**M**), ♀ holotype (**N**), face (**O**,**P**).

**Figure 5:**
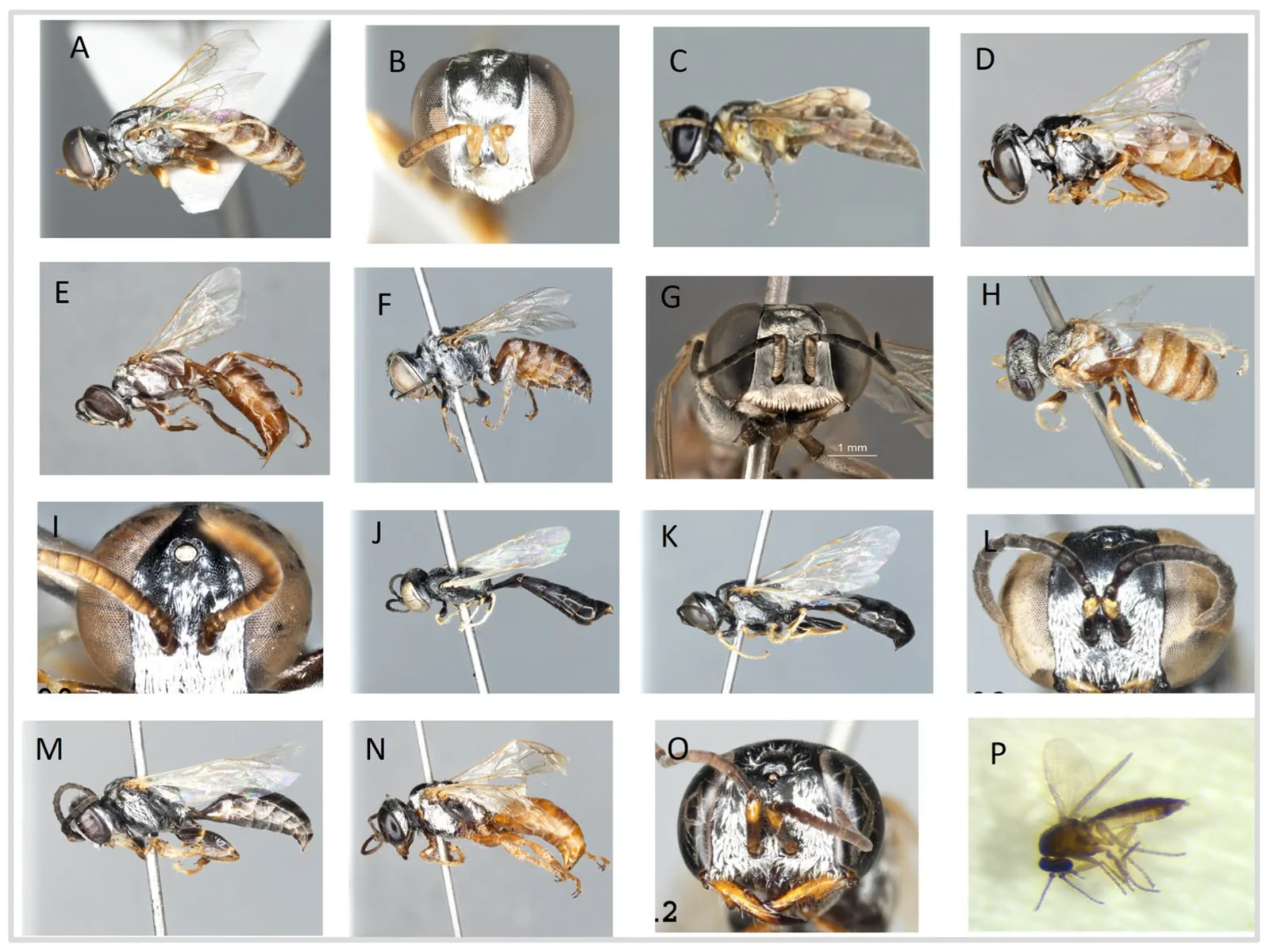
(**A**–**P**). *Gastrosericus alwathbaensis*; ♂ holotype, lateral view (**A**), face (**B**). *Gastrosericus barqaalsuqoorensis*; ♂ holotype, lateral view (**C**), ♀ holotype, lateral view (**D**). *Gastrosericus sheikhae*; ♀ holotype, lateral view (**E**), ♂ holotype, lateral view (**F**), ♂ holotype, face (**G**). *Oxybelus sajiae*; ♀ holotype, laterodorsal view (**H**), face (**I**). *Parapiagetia alwathbaensis*; ♀ holotype, lateral view (**J**), ♂ holotype, lateral view (**K**), ♂ holotype, face (**L**). *Parapiagetia huwrobertsi*; ♀ holotype (**M**), ♂ holotype, lateral view (**N**), face (**O**). *Dasyhelea* (*Dasyhelea*) *patiae*; ♀ paratype (**P**).

**Figure 6:**
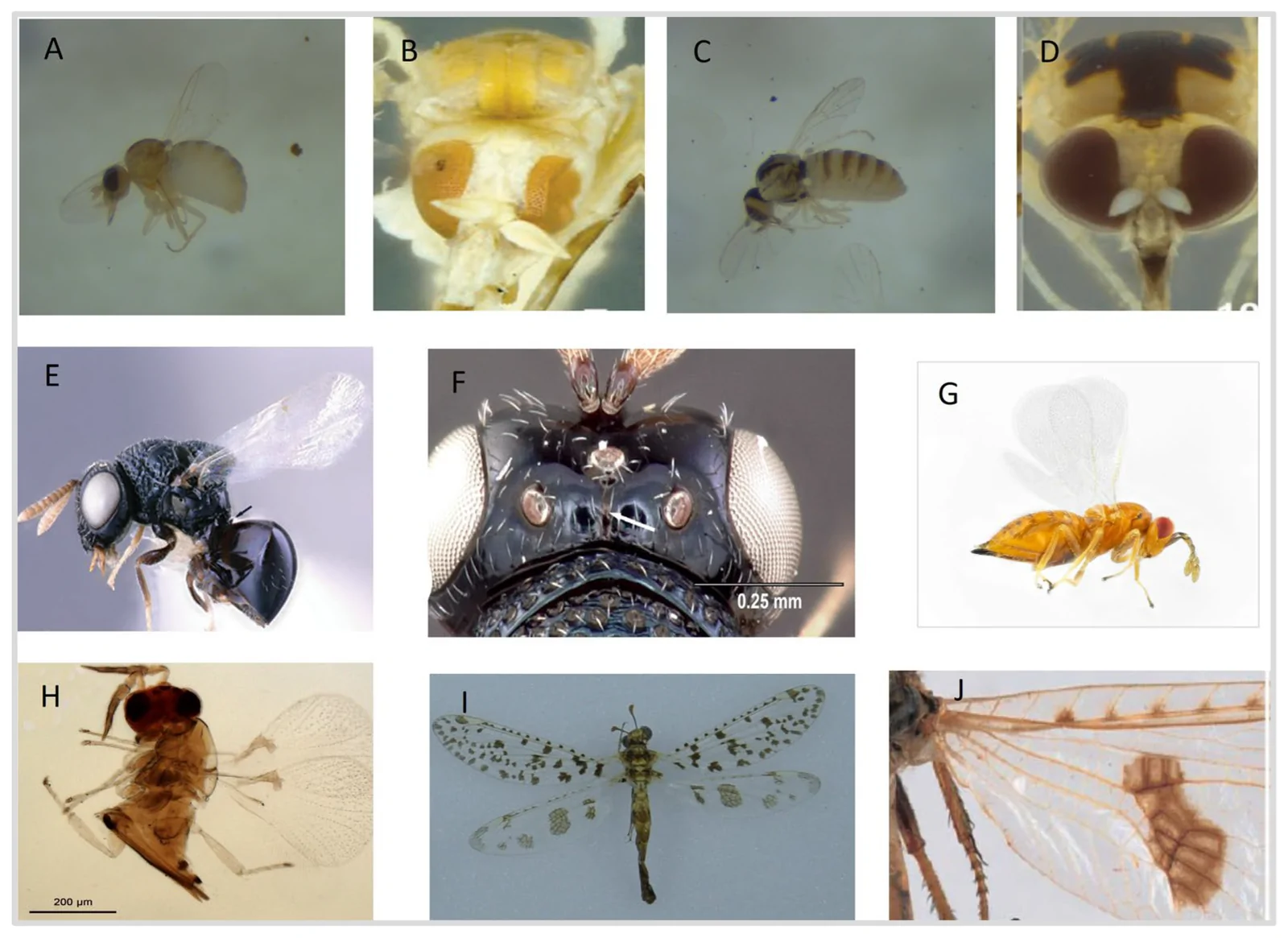
(**A**–**J**) *Leylaiya citrina*; habitus, lateral view (**A**), head and anterior thorax, frontal view (**B**). *Leylaiya medioccipitalis*; habitus, lateral view (**C**), head and anterior thorax, frontal view (**D**). *Perilampus houbarensis*; holotype, habitus, lateral view (**E**), head, dorsal view (**F**). *Epoligosita arabica*; ♀ paratype, lateral view (**G**). *Ufens curvipenis*; ♀ holotype (**H**). *Tomatarella hoelzeli*; CuP scarcely curved to the hind margin of the hind wing (**I**,**J**).

### 3.1. Updated Taxonomic Checklist of Species Formally Described from the EAD Invertebrate Inventory Project

The following checklist summarizes the insect species formally described from specimens collected through the EAD Invertebrate Inventory Project. The taxa are arranged according to their taxonomic classification, with entries presented alphabetically within the relevant taxonomic group. For each species, information on the original description, collection locality, geographic distribution, EAD voucher material, and relevant remarks are provided. The taxonomic information is based on the original published descriptions and does not constitute a new taxonomic or nomenclatural act.

#### 3.1.1. Order Hymenoptera Linnaeus, 1758

##### Family Bradynobaenidae Saussure, 1892

Bradynobaenid wasps are solitary, ground-dwelling wasps commonly associated with arid and semi-arid habitats. They are generally burrowing species whose larvae are provisioned with insect prey. The family remains relatively understudied, and records from arid regions continue to contribute to our understanding of its diversity and distribution.

1. *Macroocula sajia* Pagliano, 2008

Material examined: The species was collected from Arabian Oryx Protected Area (6), Al Khatam, Madinat Zayed, and Sultan Bin Tahnoon in the Emirate of Abu Dhabi, UAE. The type series comprises a holotype female and additional female paratypes collected using pitfall traps. The relevant EAD material is maintained in the Invertebrate Collection of the Environment Agency–Abu Dhabi (EAD-INV; voucher No. EAD-INV-003003).

Geographic distribution: United Arab Emirates; Emirate of Abu Dhabi.

Remarks: *Macroocula sajia* [13] is characterized by a predominantly yellow body, black eyes, and mandibles that are yellow basally and light brown distally. The body is covered with white pubescence approximately half the eye diameter in length, with longer lateral setae on the head, mesosoma, and metasoma. The wings are absent, and the female body length is approximately 8.5 mm. Male specimens remain unknown. Diagnostic separation from congeners is based on the combination of mandibular coloration, pubescence pattern, and metasomal morphology.

2. *Macroocula tigarae* Gadallah & Pagliano, 2014

Material examined: The species was described from material collected in the Public Hunting Triangle & Arabian Oryx Protected Area (6) in the Emirate of Abu Dhabi, UAE. The type series comprises a holotype female and additional female paratypes collected using pitfall traps. The relevant EAD material is maintained in the Invertebrate Collection of the Environment Agency–Abu Dhabi (EAD-INV; voucher No. EAD-INV-003002).

Geographic distribution: United Arab Emirates; Emirate of Abu Dhabi.

Remarks: *Macroocula tigarae* [13] is characterized by a predominantly yellow body with dense white pubescence, black eyes, and mandibles that are yellow basally and brown distally. The head is rounded, the pronotum is smooth and slightly arched, and the appendages are yellow. The wings are absent, and the female body length is approximately 8.5 mm. Male specimens were unknown in the original description. Diagnostic separation from congeners and related taxa is based on the distinctive mandibular color pattern in combination with the overall yellow body coloration and body size.

##### Family: Chrysididae Latreille, 1802

Cuckoo wasps are small, metallic, often brightly colored wasps known for their parasitoid or kleptoparasitic associations with the nests of other solitary bees and wasps. Their larvae develop using host eggs, larvae, or provisions. They are commonly encountered in sunny habitats where suitable host nests occur. Their strongly sclerotized exoskeleton provides protection from host defensive behavior. Cuckoo wasps are an important component of terrestrial ecosystems and contribute to the regulation of host populations.

1. *Hedychridium anithaae* Strumia, 2014

Material examined: The species was described from material collected in the AWWR (1) in the Emirate of Abu Dhabi, UAE, using water traps. The type series comprises a holotype female, three male paratypes, and one female paratype. The relevant EAD material is maintained in the Invertebrate Collection of the Environment Agency–Abu Dhabi (EAD-INV; voucher No. EAD-INV-003004).

Geographic distribution: United Arab Emirates; Emirate of Abu Dhabi.

Remarks: *Hedychridium anithaae* [14] is characterized by a brilliant metallic green body with iridescent blue and purple highlights, black eyes, and dark metallic green antennae. The head is rounded and densely punctate, while the pronotum and mesoscutum are finely punctate. The metasoma is ovate and densely punctate, with bluish-green coloration. The legs are slender and metallic, with darker tarsi, and the wings are hyaline. The female body length is approximately 5–6 mm. Diagnostic separation from congeners is based on morphometric ratios in combination with distinctive integumental coloration. The species was originally described from material collected in a wetland habitat within a protected area.

2. *Hedychridium caerulescens* Strumia & van Harten, 2020

Material examined: The species was described from specimens collected in the Al Tawi Protected Area (5) and Al Bida’a Protected Area (3) in the Emirate of Abu Dhabi, UAE. The type series comprises a holotype female collected using a Malaise trap at Al Tawi Protected Area (5) and two paratypes. The relevant EAD material is maintained in the Invertebrate Collection of the Environment Agency–Abu Dhabi (EAD-INV; voucher Nos. EAD-INV-003005–EAD-INV-003006).

Geographic distribution: United Arab Emirates; Emirate of Abu Dhabi.

Remarks: *Hedychridium caerulescens* [15] is characterized by its small body size (3.9–4.1 mm), metallic blue-green head and mesosoma, and distinctive metasomal coloration. Morphological characters include a finely striated scapal basin, mandibles with an inner tooth, a finely punctate mesonotum, and a narrow transparent apical rim on T3. Diagnostic separation from congeners is based on the combination of metasomal coloration, mandibular structure, and integumental sculpture. The present account summarizes previously published taxonomic information and does not constitute a new taxonomic or nomenclatural act.

3. *Chrysis houbaraeensis* Franco Strumia & Antonius van Harten, 2020

Material examined: The species was described from specimens collected in the Al Houbara Protected Area (2) in the Emirate of Abu Dhabi, UAE, using a Malaise trap. The type series comprises a holotype male collected by A. Saji and A. van Harten and three paratypes from the same locality. The relevant EAD material is maintained in the Invertebrate Collection of the Invertebrate Collection of the Environment Agency–Abu Dhabi (EAD-INV; voucher No. EAD-INV-003007).

Geographic distribution: United Arab Emirates; Emirate of Abu Dhabi.

Remarks: *Chrysis houbaraeensis* [15] is characterized by a metallic green-blue head, a metallic metasoma with a bluish distal hue, and short, silky white setae. The integument is finely punctate, the antennae are robust, and the legs are non-metallic pale brown. The first hind tarsal segment is reduced, and the wings are hyaline with light brown veins. Diagnostic separation from congeners is based on the combination of metasomal coloration, the reduced first hind tarsal segment, non-metallic legs, and fine integumental punctation. The present account summarizes previously published taxonomic information and does not constitute a new taxonomic or nomenclatural act.

##### Family: Crabronidae Latreille, 1802

Digger wasps are solitary, ground-nesting wasps that excavate burrows and provision with their nests with paralyzed insects or spiders for their larvae. They display diverse hunting and nesting behaviors and are widely distributed across various habitats. Despite their ecological significance, many species remain undescribed, with new discoveries continuing to expand knowledge of the family occur across a wide range of habitats.

1. *Ammatomus wathabensis* Schmid-Egger, 2019

Material examined: The species was described from material collected at the AWWR (1) in the Emirate of Abu Dhabi, UAE. The type series includes a paratype female collected using a Malaise trap and the relevant EAD material is deposited in the Invertebrate Collection of the Environment Agency–Abu Dhabi (EAD-INV; voucher No. EAD-INV-003011).

Geographic distribution: United Arab Emirates; AWWR (1), Emirate of Abu Dhabi.

Remarks: *Ammatomus wathabensis* [16] is characterized by a robust body (♀ female 7–9.5 mm) with a distinctive coloration pattern combining combination of yellow, black, and reddish-brown tones coloration. The head is broad, with dark antennae and compound eyes. The forelegs bear comb-like digging structures, the thorax is moderately convex with contrasting coloration; and the wings are hyaline with slight infuscation; and the elongate abdomen lacks the transparent basal lamella typical characteristic of related congeners. Males are slightly smaller (~approximately 8.5 mm), similarly colored, and slenderer. Diagnostic separation from congeners is based on the absence of the transparent, leaf-like structure on the first abdominal segment and the unique distinctive combination of body coloration. The specific epithet wathabensis refers to the type locality, AWWR (1). The species is restricted to AWWR (1), highlighting the ecological and conservation significance of this protected area. The specific epithet wathabensis refers to the type locality, adjective in the nominative singular.

2. *Oryttus konradschmidti* Schmid-Egger, 2014

Material examined: The species was described from material collected at the AWWR (1) in the Emirate of Abu Dhabi, UAE. The type series comprises a holotype male collected in using a Malaise trap by A. Saji &and A. van Harten. As the physical specimen is not currently deposited in the EAD Invertebrate Collection of the EAD, the voucher record is represented by photographs (EAD; voucher No. EAD-INV-003012).

Geographic distribution: Tunisia and the United Arab Emirates; AWWR (1), Emirate of Abu Dhabi.

Remarks: *Oryttus konradschmidti* [17] is characterized by a bright red body and a body length of approximately 9.8 mm (♂in the male holotype). The head is coarsely punctate; the antennae are modified, with antennal segments VIII–XIII bearing tyloids and a ventral keel. The mesosoma has coarse, dense punctation, while the propodeum is laterally comb-like with a finely sculptured dorsum. The metasoma shows has coarse punctation on tergite II and finer punctation on the remaining tergites; sternite I bear a medial keel, and sternite VIII is deeply V-emarginate. Females are larger (~approximately 10.5 mm), with an entirely red face, lighter inner eye margin, fore tarsi with digging spines, and a flat pygidium with a sharp rim. Diagnostic separation from congeners is based on the combination of bright red coloration and genitalia structure. The species is known from Tunisia and AWWR (1) in the Emirate of Abu Dhabi, UAE. The specific epithet konradschmidti honors Konrad Schmidt, a specialist in Sphecid wasps, who first recognized it as an undescribed taxon; proper noun in apposition.

3. *Didineis mokrousovi* Schmid-Egger, 2022

Material examined: The species was described from specimens collected in AWWR (1) and Al Bida’a Protected Area, (3) in the Emirate of Abu Dhabi, UAE. The type series comprises paratypes including one male and one female from each locality; the relevant EAD material is deposited in the Invertebrate Collection of the Environment Agency–Abu Dhabi (EAD-INV; voucher Nos. EAD-INV-003013–EAD-INV-003014).

Geographic distribution: United Arab Emirates, Emirate of Abu Dhabi recorded from AWWR (1) and Al Bida’a Protected Area (3).

Remarks: *Didineis mokrousovi* [18] is characterized by a black body with distinct yellow markings. Males measure approximately 8.0 mm in length and possess short, thickened antennal flagellomeres. The elytra are black with yellow markings. Diagnostic separation from congeners is based on the unique combination of male antennal morphology and distinctive body coloration. The specific epithet mokrousovi honors Mikhail Mokrousov, a specialist in aculeate wasps.

4. *Gastrosericus albidaaensis* Schmid-Egger, 2022

Material examined: The species was described from specimens collected in AWWR, (1), Al Houbara Protected Area, (2), Al Bida’a Protected Area, (3), Umm Al Quwain, Wadi Wurayah, and Barqa Al Saqoor (4) in the Emirate of Abu Dhabi. The type series comprises a holotype female and multiple paratypes and the relevant EAD material is deposited in the Invertebrate Collection of the EAD (EAD-INV; voucher Nos. EAD-INV-003015–EAD-INV-003018).

Geographic distribution: United Arab Emirates, including localities in the Emirate of Abu Dhabi, Umm Al Quwain, and Wadi Wurayah.

Remarks: *Gastrosericus albidaaensis* [18] is characterized by a predominantly black body with conspicuous red markings. Females measure approximately 9.2 mm in length, while males remain unknown. Diagnostic separation from congeners is based on the distinctive coloration pattern and female body size. The specific epithet albidaaensis refers to Al Bida’a Protected Area, (3), one of the principal localities from which the species was collected.

5. *Gastrosericus alwathbaensis* Schmid-Egger, 2022

Material examined: The species was described from specimens collected in the AWWR, (1) in the Emirate of Abu Dhabi, UAE. The type series comprises a holotype male and the relevant EAD material is deposited in the Invertebrate Collection of the Environment Agency–Abu Dhabi (EAD-INV; voucher No. EAD-INV-003019).

Geographic distribution: United Arab Emirates, Emirate of Abu Dhabi; currently recorded from AWWR (1).

Remarks: *Gastrosericus alwathbaensis* [18] is characterized by a black body with distinct ivory-white bands on the tergites. The male measures approximately 4.1 mm in length and possesses a short marginal wing cell and deeply impressed sterna. Females remain unknown. Diagnostic separation from congeners is based on the unique combination of tergal coloration, short marginal wing cell, and deeply impressed male sterna. The specific epithet alwathbaensis refers to the type locality, AWWR (1).

6. *Gastrosericus barqaalsuqoorensis* Schmid-Egger, 2022

Material examined: The species was described from specimens collected in Barqa Al Saqoor (4) and Al Ajban in the Emirate of Abu Dhabi, UAE. The type series comprises a holotype female and a male specimen and the relevant EAD material is deposited in the Invertebrate Collection of the Environment Agency–Abu Dhabi (EAD-INV; voucher No. EAD-INV-003020).

Geographic distribution: United Arab Emirates, Emirate of Abu Dhabi; recorded from Barqa Al Saqoor (4) and Al Ajban.

Remarks: *Gastrosericus barqaalsuqoorensis* [18] exhibits marked sexual dimorphism in coloration. Females measure approximately 7.0 mm in length and possess a black body with a yellow head and mesosoma and a bright red metasoma, whereas males measure approximately 5.0 mm in length and are greyish black with dense yellow-silvery pubescence. Diagnostic separation from related species is based on the distinctive sexual coloration pattern and body size. The specific epithet barqaalsuqoorensis refers to the type locality, Barqa Al Saqoor.

7. *Gastrosericus sheikhae* Schmid-Egger, 2022

Material examined: The species was described from specimens collected in Barqa Al Saqoor (4), AWWR (1), Al Houbara Protected Area (2), Al Bida’a Protected Area (3), and Yaw Al Dibsa Protected Area (9). The type series comprises a holotype female and multiple paratypes and the relevant EAD material is deposited in the Invertebrate Collection of the Environment Agency–Abu Dhabi (EAD-INV; voucher Nos. EAD-INV-003021–EAD-INV-003024).

Geographic distribution: United Arab Emirates, Emirate of Abu Dhabi; recorded from Barqa Al Saqoor (3), AWWR (1), Al Houbara Protected Area (2), Al Bida’a Protected Area (3), and Yaw Al Dibsa Protected Area (9).

Remarks: *Gastrosericus sheikhae* [18] exhibits pronounced sexual dimorphism. Females measure approximately 11.0 mm in length and are predominantly orange-red with a black head, antennae, and mesonotum, whereas males measure 7.0–9.0 mm in length and possess a red head and mesosoma. Diagnostic separation from related species is based on the distinctive sexual coloration pattern and body size. The specific epithet sheikhae honors H.E. Dr. Shaikha Salem Al Dhaheri, Secretary General of the EAD.

8. *Oxybelus sajiae* Schmid-Egger, 2022

Material examined: The species was described from specimens collected in the Al Houbara Protected Area (2) and Al Bida’a Protected Area, (3) in the Emirate of Abu Dhabi, United Arab Emirates. The type series comprises a holotype female and three female paratypes and the relevant EAD material is deposited in the Invertebrate Collection of the Environment Agency–Abu Dhabi (EAD-INV; voucher Nos. EAD-INV-003025–EAD-INV-003026).

Geographic distribution: United Arab Emirates, Emirate of Abu Dhabi; recorded from Al Houbara Protected Area (2) and Al Bida’a Protected Area (3).

Remarks: *Oxybelus sajiae* 18] is characterized by a reddish pronotum, propodeum, legs, and metasoma with distinct white markings, and yellowish mandibles with a black apex. Females measure approximately 5.9 mm in length, whereas males remain unknown. Diagnostic separation from congeners is based on the unique combination of coloration of the pronotum, propodeum, legs, metasoma, and mandibles. The specific epithet sajiae honors Dr. Anitha Saji for her contributions to project coordination and specimen collection.

9. *Parapiagetia alwathbaensis* Schmid-Egger, 2022

Material examined: The species was described from specimens collected in AWWR (1) in the Emirate of Abu Dhabi, UAE. The type series comprises a holotype male and multiple paratypes and the relevant EAD material is maintained and deposited in the Invertebrate Collection of the Environment Agency–Abu Dhabi (EAD-INV; voucher No. EAD-INV-003027).

Geographic distribution: United Arab Emirates, Emirate of Abu Dhabi; currently recorded from AWWR (1).

Remarks: *Parapiagetia alwathbaensis* [18] is characterized by a black body with distinct yellowish-white markings. Males measure approximately 7.2 mm in length, whereas females range from 7.0 to 8.5 mm. Diagnostic separation from congeners is based on the unique combination of body coloration and body size. The specific epithet alwathbaensis refers to the type locality, AWWR (1).

10. *Parapiagetia huwrobertsi* Schmid-Egger, 2022

Material examined: The species was described from specimens collected in Al Houbara Protected Area (2), AWWR (1), Al Ajban, Al Bida’a Protected Area (3), Barqa Al Suqoor Protected Area, (4), and Jabal Hafit National Park (8) in the Emirate of Abu Dhabi, UAE. The type series comprises a holotype female and numerous paratypes and the relevant EAD material is maintained and deposited in the Invertebrate Collection of the Environment Agency–Abu Dhabi (EAD-INV; voucher Nos. EAD-INV-003028–EAD-INV-003032).

Geographic distribution: United Arab Emirates, Emirate of Abu Dhabi.; recorded from Al Houbara Protected Area (2), AWWR (1), Al Ajban, Al Bida’a Protected Area (3), Barqa Al Suqoor Protected Area (4), and Jabal Hafit National Park (8).

Remarks: *Parapiagetia huwrobertsi* [18] exhibits marked sexual dimorphism. Females measure approximately 10.5 mm in length and are predominantly black with red mandibles, pronotal lobe, legs, and metasoma, whereas males measure 6.0–7.2 mm in length and possess yellowish markings on the mandibles, scape, tegula, and legs, with dense silvery pubescence on the face and mesosoma. Diagnostic separation from congeners is based on the entirely red female metasoma, clypeal tooth placement, and the uniformly greyish abdomen and femora of males. The specific epithet huwrobertsi honors entomologist Huw Roberts for his contributions to the collection and documentation of UAE wasps.

##### Family: Gasteruptiidae Ashmead, 1900

1. *Gasteruption alwathbaense* Saure et al., 2017

Gasteruptiid wasps are a relatively small group of parasitoid wasps primarily associated with solitary bees. Females lay their eggs in host nests, where the developing larvae consume the host egg or larva and, in some species, the stored provisions. The family is cosmopolitan, with its highest diversity reported from Australia. Adults are free-living and feed mainly on nectar and occasionally pollen, often visiting flowers with readily accessible nectar. They are readily recognized by their elongated cervical region, swollen hind femora, and slender abdomen inserted high on the thorax.

Material examined: The species was described from material collected in the AWWR (1) in the Emirate of Abu Dhabi, UAE, using a Malaise trap. The type series comprises a holotype female. The relevant EAD material is maintained in the Invertebrate Collection of the Environment Agency–Abu Dhabi (EAD-INV; voucher No. EAD-INV-003008).

Geographic distribution: United Arab Emirates; Emirate of Abu Dhabi.

Remarks: *Gasteruption alwathbaense* [19] is characterized by a relatively large body size (11.3 mm) and a very long ovipositor sheath (16.2 mm). The head is elongate with a distinctly long cervical region. The mesoscutum is smooth and polished, with irregular deep pit-like punctures, while the pronotum and mesopleuron are finely sculptured. The hind femora are swollen, and the metasoma is elongate and inserted high on the propodeum. The ovipositor is long and projects well beyond the metasoma. The wings are hyaline with faint venation. Male specimens were unknown in the original description. Diagnostic separation from congeners is based on the combination of a smooth, pit-punctate mesoscutum, elongated cervical region, swollen hind femora, and high metasomal insertion. The species is associated with wetland and oasis habitats in the United Arab Emirates.

##### Family Ichneumonidae Latreille, 1802

Ichneumonidae are parasitoid wasps characterized by slender bodies, long antennae, two pairs of membranous wings, and a distinct constriction between the thorax and abdomen. Females typically possess a well-developed ovipositor, and most species are parasitoids of other insects.

1. *Atropatopsis abudhabiensis* Sudheer & Narendran, 2007

Material examined: The species was originally described from material collected in the Al Dhafra Desert, in the Emirate of Abu Dhabi, UAE, using pitfall traps. The type series comprises a female holotype and two female paratypes. Relevant EAD material is maintained in the Invertebrate Collection of the Environment Agency–Abu Dhabi (EAD-INV; voucher No. EAD-INV-003001).

Geographic distribution: United Arab Emirates; Emirate of Abu Dhabi.

Remarks: *Atropatopsis abudhabiensis* [12] was described as a new species concurrently with the establishment of the genus *Atropatopsis* Sudheer & Narendran [12]. The species is characterized by a predominantly black body, yellowish to dark antennae, red ocelli, and dense, short, silvery-white setation. The diagnostic characters and separation from related taxa are based on the morphological features provided in the original description, including body coloration, integumental sculpture, and female genital characters. Male specimens were not known at the time of the original description (Figure 4A).

##### Family: Pompilidae Latreille, 1805

Spider-hunting wasps are solitary wasps specialized in hunting and paralyzing spiders, which they use to provision their nests. Members of the family exhibit considerable morphological diversity and possess adaptations for locating, capturing, and transporting spider prey. They are widely distributed and play an important ecological role as predators that contribute to the regulation of spider populations in terrestrial ecosystems.

1. *Ferreola alraeesii* Schmid-Egger & Al-Jahdhami, 2018

Material examined: The species was described from material collected in the AWWR (1) in the Emirate of Abu Dhabi, UAE, and with additional sites material from localities in Oman. The type series comprises a holotype female collected using a Malaise trap AWWR; the relevant EAD material is maintained in the EAD; voucher No. EAD-INV-003010) and paratypes including comprising males and females from the same locality AWWR and Oman, collected on various different dates using Malaise traps. Geographic distribution: United Arab Emirates; Emirate of Abu Dhabi and Oman. Within the Emirate of Abu Dhabi, the species is known from the AWWR (1).

Remarks: *Ferreola alraeesii* [20] is characterized by a robust body with striking red and black coloration. Females are relatively large (18–20 mm), with a rounded head bearing dense black pubescence, dark antennae, large black eyes, an elongated pronotum distinctly constricted at midlength in dorsal view, long spiny legs, and darkened wings with subtle iridescence. Males are smaller (9–12 mm) and similarly colored but slightly slenderer. Diagnostic separation from congeners is based on the combination of large body size, pronotal morphology, coloration, and robust body form, which is distinguishes the species among Palaearctic Ferreola. The species is associated with wetland habitats and agile exhibits predatory behavior. Its known distribution includes AWWR (1), in the Emirate of Abu Dhabi, UAE, and selected localities in Oman. Its behavior is characteristic of pompilid wasps. The specific epithet alraeesii honors a distinguished the Omani entomologist Ali Ahmed Al-Raeesi for his contributions to insect taxonomy and education; noun in the genitive case.

##### Perilampidae Latreille, 1809

Perilampid wasps are a small but distinctive group of chalcidoid wasps, many of which are hyperparasitoids, developing on other parasitic parasitoid wasps or flies. Adults are often recognized by their robust bodies and metallic coloration and robust bodies, while their larvae exhibit unusual hypermetamorphic development. Although relatively uncommon in collections, perilampids are ecologically significant parasitoids, and new records contribute to our understanding of their diversity, trophic interactions, and distribution.

1. *Perilampus houbarensis* Yoo and& Darling, 2021

Material examined: The species was described from specimens collected in Al Houbara Protected Area, (2) in the Emirate of Abu Dhabi, UAE. The type series comprises a holotype and male paratypes deposited. The relevant EAD material is maintained in the Invertebrate Collection of the Environment Agency–Abu Dhabi (EAD-INV; voucher No. EAD-INV-003039).

Geographic distribution: United Arab Emirates; Emirate of Abu Dhabi.

Remarks: *Perilampus houbarensis* [21] is characterized by a predominantly black body with iridescent blue reflections on the metasoma and dorsellum. The legs are predominantly brown to black, and the antennae are brown. Diagnostic separation from congeners is based particularly on the distinctive male coloration and the characteristic reflective iridescent blue patterning of the metasoma and dorsellum. Female specimens remain currently unknown. The specific epithet houbarensis refers to the type locality, Al Houbara Protected Area (2), from which the species was described.

##### Family: Trichogrammatidae Haliday, 1833

Trichogrammatid wasps are among the smallest parasitoid wasps, with many species measuring less than 1 mm in body length. They are primarily egg parasitoids of Lepidoptera and other insects, therefore making them ecologically and economically important components of natural biocontrol agents. The family is widely distributed and taxonomically diverse, with new species continuing to be discovered. However, the minute size of its members poses challenges for species identification and biological research. New species and records continue to be described worldwide, expanding the knowledge of trichogrammatid diversity and distribution.

1. *Epoligosita arabica* Gennaro Viggiani, 2021

Material examined: The species was described [22] from material collected at the Al Houbara Protected Area (2) in the Emirate of Abu Dhabi, UAE. The type series comprises a holotype female collected using a Malaise trap by A. Saji and A. van Harten, together with additional female paratypes from the same locality. The relevant EAD material is deposited in the Invertebrate Collection of the Environment Agency–Abu Dhabi (EAD-INV; voucher No. EAD-INV-003040).

Geographic distribution: United Arab Emirates; Emirate of Abu Dhabi, currently known only from Houbara Protected Area (2).

Remarks: *Epoligosita arabica* is a minute, species measuring approximately 0.5–0.8 mm in body length. It is predominantly yellow, with a yellow general coloration, dusky areas on the thorax, and brown lateral patches on the abdominal tergites. Diagnostic characters include distinctive forewing venation and female genital morphology, which separate it from related species of Epoligosita. Male specimens are currently unknown. The species is currently known only from the Al Houbara Protected Area (2) in the Emirate of Abu Dhabi Emirate, UAE. The specific epithet arabica refers to the Arabian Peninsula and is treated as an adjective in apposition. The species is currently known only from Al Houbara Protected Area (2) in the Emirate of Abu Dhabi, United Arab Emirates.

2. *Ufens curvipenis* Gennaro Viggiani, 2021

Material examined: The species was described from material collected in AWWR (1), in the Emirate of Abu Dhbai, UAE. The type series comprises a holotype female collected using a Malaise trap by A. Saji and A. van Harten, along with additional female paratypes from the same locality. The relevant EAD material is deposited in the Invertebrate Collection of the Environment Agency–Abu Dhabi (EAD-INV; voucher No. EAD-INV-003041).

Geographic distribution: United Arab Emirates; Emirate of Abu Dhabi, currently known only from AWWR (1).

Remarks: *Ufens curvipenis* [22] is a minute species, measuring approximately 0.5–0.8 mm in body length. The body is predominantly yellow, with dusky thoracic areas on the thorax and small brown lateral patches on the abdominal tergites. Diagnostic characters include separation from related species based on distinctive forewing venation in females, while males are characterized by a distinctive curved aedeagus. However, as the available type material comprises females and male specimens are currently unknown, the male genital character should not be presented as an observed character of the type series unless supported by the original description. The specific epithet curvipenis is derived from Latin and refers to the curved form of the male genitalia; it is treated as an adjective in apposition. The species is currently known only from AWWR (1), in the Emirate of Abu Dhabi, UAE.

#### 3.1.2. Order: Diptera Linnaeus, 1758

##### Family: Ceratopogonidae Newman, 1834

Biting midges are minute flies occurring in a wide range of aquatic, semi-aquatic, and terrestrial habitats. Although many ceratopogonids are known for blood-feeding behavior, species of the genus Dasyhelea are non-biting and are generally associated with nectar feeding as adults. Larvae of *Dasyhelea* commonly develop in moist, aquatic, or semi-aquatic substrates. The genus is cosmopolitan and species diversity remains incompletely documented in several regions.

1. *Dasyhelea* (*Dasyhelea*) *patiae* Szadziewski & Gwizdalska-Kentzer, 2020

Material examined: The species was described from specimens collected in the AWWR (1), in the Emirate of Abu Dhabi, UAE. The type series comprises a holotype male and a paratype male. The relevant EAD material is maintained in the Invertebrate Collection of the Environment Agency–Abu Dhabi (EAD-INV; voucher No. EAD-INV-003033).

Geographic distribution: United Arab Emirates; Emirate of Abu Dhabi.

Remarks: *Dasyhelea patiae* [23] is characterized by a dark brown body, yellow scutellum, dark halteres, pale legs with the fifth tarsomere dark brown, and yellowish abdominal pleurae. The male has pubescent eyes and elongate distal antennal flagellomeres. The apicolateral processes of abdominal tergite 9 are greatly reduced, while the parameres are short and broad and bear a few apical setae. Females are currently unknown. Diagnostic separation from congeners is based on the combination of antennal morphology, the structure of abdominal tergite 9 and parameres, and the distinctive body coloration. The specific epithet patiae honors Patrycja Dominiak for her contributions to the study of Palaearctic Ceratopogonidae. The species was recorded from the wetland environment of AWWR (1). The present account summarizes previously published taxonomic information and does not constitute a new taxonomic or nomenclatural act.

##### Hybotidae Meigen, 1820

Hybotid flies are small, predatory Diptera with compact-to-elongated bodies, large compound eyes, short aristate antennae, and often-arched thoraces. Their legs are generally elongated and adapted for rapid terrestrial movement, while the wings are typically hyaline with reduced venation. Hybotids are active predators of small arthropods and occur in diverse habitats, including wetlands, riparian zones, and arid environments, where they contribute to the regulation of invertebrate populations.

1. *Drapetis wathabiensis* (manuscript name; unpublished)

Taxonomic status: *Drapetis wathabiensis* is currently an unpublished taxon represented by material collected during the EAD Invertebrate Inventory Project. A separate taxonomic treatment of this taxon is currently in preparation. The name is therefore used here only as a manuscript name and does not constitute a formally available species name or a new nomenclatural act under the ICZN.

Material examined: The specimens were collected from the AWWR (1), in the Emirate of Abu Dhabi, UAE, using Malaise traps. The holotype male was collected between 1 and 30 April 2015 by A. Saji and A. van Harten. The relevant EAD material is preserved in 80% ethanol and deposited in the Invertebrate Collection of the Environment Agency–Abu Dhabi (EAD-INV; voucher No. EAD-INV-003009).

The paratype series comprises 100 males and 100 females collected from the same locality between January and May 2015 using Malaise traps by A. Saji and A. van Harten. The specimens are preserved in ethanol and stored in 2 mL micro-cryovials.

Geographic distribution: currently known from AWWR (1), United Arab Emirates; Emirate of Abu Dhabi.

Remarks: The taxon is represented by extensive male and female material collected during the monitoring programme. Paratypes are deposited in the EAD Invertebrate Collection (EAD-INV), Abu Dhabi; Senckenberg Deutsches Entomologisches Institut (SDEI), Müncheberg, Germany; Royal Belgian Institute of Natural Sciences (RBINS), Brussels, Belgium; and the private Diptera collection of Andreas Stark (ASPC), Germany. A formal taxonomic treatment is in preparation. Accordingly, *Drapetis wathabiensis* is included in the present checklist to document the taxonomic material generated by the long-term monitoring programme, but it is not counted as a formally described species and no nomenclatural act is proposed here. The taxon is included here as part of the historical synthesis of insect diversity documented through the EAD Invertebrate Inventory Project. Its formal taxonomic and nomenclatural status will be updated following publication of the corresponding taxonomic description.

##### Family: Mythicomyiidae Melander, 1906

Micro-bee flies are minute members of the superfamily Bombylioidea, with many species measuring less than 2 mm in body length. They are particularly associated with arid and semi-arid environments, although their biology remains poorly known for many species. They are generally considered parasitoids of other insects. Despite their extremely small size, mythicomyiids exhibit considerable morphological and taxonomic diversity, and new species continue to be described from poorly studied regions.

1. *Leylaiya citrina* Evenhuis & Gharali, 2020

Material examined: The species was described from specimens collected in Al Bida’a Protected Area (3), AWWR (1), and Barqa Al Suqoor Protected Area (4) in the Emirate of Abu Dhabi, UAE. The relevant EAD material is maintained in the Invertebrate Collection of the Environment Agency–Abu Dhabi (EAD-INV; voucher Nos. EAD-INV-003034–EAD-INV-003036).

Geographic distribution: United Arab Emirates; Emirate of Abu Dhabi.

Remarks: *Leylaiya citrina* [24] is characterized by a uniformly yellowish-white body, with the occiput distinctly yellow to yellowish orange. Males measure approximately 0.9–1.3 mm in body length, with a wing length of 0.8–1.2 mm. Females are currently unknown. Diagnostic separation from congeners is based on the combination of its extremely small body size, pale yellowish-white coloration, and distinctive yellow to yellowish-orange occipital pigmentation. The specific epithet citrina is derived from the Latin word citrinus, meaning “lemon yellow”, referring to the characteristic body coloration. The species has been recorded from protected wetland habitats in the Emirate of Abu Dhabi.

2. *Leylaiya medioccipitalis* Evenhuis & Gharali, 2020

Material examined: The species was described from material collected in Al Bida’a Protected Area (3) and Al Tawi Protected Area (5), in the Emirate of Abu Dhabi, UAE. The type series comprises male paratypes collected using Malaise traps by A. van Harten. The relevant EAD material is maintained in the Invertebrate Collection of the Environment Agency–Abu Dhabi (EAD-INV; voucher Nos. EAD-INV-003037–EAD-INV-003038).

Geographic distribution: United Arab Emirates; Emirate of Abu Dhabi.

Remarks: *Leylaiya medioccipitalis* [24] is characterized by a yellow body with a distinct black-to-brown median marking on the occiput and darker sclerotization of the lower pleural sclerites. Males measure approximately 1.4–1.7 mm in body length. Females are currently unknown. Diagnostic separation from *Leylaiya citrina* and other congeners is based particularly on the distinctive median occipital marking and the coloration of the pleural sclerites. The species is known from Al Bida’a Protected Area (3) and Al Tawi Protected Area (5) in the Emirate of Abu Dhabi, UAE. The specific epithet medioccipitalis is derived from the Latin medius (“middle”) and occiput (“back of the head”), referring to the distinctive median marking on the occiput.

#### 3.1.3. Order: Neuroptera

##### Family: Myrmeleontidae

1. *Tomatarella hoelzeli* Ábrahám, 2014

Antlions are neuropteran insects particularly well known for the predatory behavior of their larvae. In many species, larvae construct conical pitfall traps in loose sandy substrates to capture ants and other small arthropods. Adults are generally weak fliers and, in many species, are active during the evening or at night. The family is diverse and widely distributed, with particularly high representation in arid and sandy environments. Material examined: The species was described from material collected at multiple localities in the United Arab Emirates, including Al-Ajban and Arabian Oryx Protected Area (6), Abu Dhabi Emirate. The type series comprises a holotype male collected in 2006 using a light trap by A. van Harten, together with multiple female paratypes from the same and additional localities. The relevant EAD specimens are maintained in the Invertebrate Collection of the Environment Agency–Abu Dhabi (EAD-INV; voucher No. EAD-INV-003042).

Geographic distribution: United Arab Emirates and the Arabian Peninsula, including localities within the Emirate of Abu Dhabi.

Remarks: *Tomatarella hoelzeli* [25] is a desert-adapted antlion characterized by long, clubbed antennae and elongated wings with distinct dark markings. The larvae are fossorial and possess large, sickle-shaped mandibles adapted for capturing small arthropod prey. The species can be distinguished from *Tomatarella markli* by its more conspicuous hindwing markings and comparatively narrower wings.

The species has been recorded from several localities in the Arabian Peninsula, including protected areas in the Emirate of Abu Dhabi such as the Arabian Oryx Protected Area (6). Its occurrence in protected desert habitats highlights the importance of these areas for documenting and conserving specialized desert insect fauna.

Spatial distribution of newly recorded species: The number of newly recorded insect species varied considerably among the surveyed protected areas (Figure 1). The highest number of species was recorded at Al Wathba Wetland Reserve (1), with 15 species, followed by Al Houbara Protected Area (2) and Al Bida’a Protected Area (3), with seven species each. Barqa Al Suqoor Protected Area (4) yielded five species, while Al Tawi Protected Area and Arabian Oryx Protected Area (6) each yielded two species. A single species was recorded from Al Dhafra Desert, Jabal Hafit National Park (8), and Yaw Al Dibsa Protected Area (9) each (Figure 7). These differences should be interpreted in the context of sampling coverage and effort, as lower numbers of newly recorded species at some sites may partly reflect reduced sampling intensity rather than lower overall insect diversity. Overall, the results demonstrate spatial variation in the occurrence of newly recorded taxa, with several protected areas contributing substantially to the overall number of species documented.

## 4. Discussion

The present study highlights the exceptional yet underexplored insect biodiversity within the terrestrial protected areas (TPAs) of the Emirate of Abu Dhabi. Among the taxa documented through these surveys are 26 insect species and one genus, representing 13 families across Hymenoptera, Diptera, and Neuroptera. Of these 26 species, 25 have been formally described in previous taxonomic publications, whereas one Drapetis taxon remains unpublished and is therefore not treated as an available nomenclatural act under the ICZN. The occurrence of these taxa across a range of habitats underscores the ecological uniqueness of the arid region and demonstrates the importance of long-term, systematic inventory efforts in documenting previously unrecognized faunal diversity. Most of these taxa were recorded from terrestrial protected areas, emphasizing their importance for conserving and documenting the Emirate’s insect biodiversity.

Despite these valuable findings, the insect specimens collected through the EAD’s Insect Inventory Project are curated and housed in the scientific reference collections of the Environment Agency–Abu Dhabi (EAD). These valuable specimens constitute an important repository of the UAE’s insect biodiversity, supporting taxonomic research, biodiversity assessments, species conservation, and long-term environmental monitoring. The collection provides a critical scientific resource for documenting the nation’s fauna and facilitating future research on the diversity and distribution of insects in the United Arab Emirates.

A comprehensive species inventory of insects within UAE protected areas will require long-term, multi-year sampling. This necessity stems from considerable interannual and seasonal fluctuations in environmental conditions, species emergence patterns, and migratory behaviors, all of which affect species detectability and population density. Furthermore, many insect groups, especially those with small body size, nocturnal or cryptic habits, or brief adult life spans, remain significantly under-documented. Wide-ranging surveys across diverse habitat types from sand dunes and gravel plains to wetlands, rocky mountain terrains, and ghaf forests using a suite of standardized collection methods (e.g., Malaise traps, pitfall traps, light traps, yellow pan traps, baited traps) are essential to improve baseline biodiversity datasets. These methods increase the likelihood of capturing taxa with highly specific microhabitat preferences or low population densities. Our findings are consistent with global trends indicating that protected areas, although central to biodiversity conservation, often fail to fully capture the diversity of insects. A previous study revealed that 76% of insect species globally are not adequately covered by existing PAs, and nearly 1900 species lack any recorded presence within protected networks [26]. Additionally, conservation planning often overlooks vital ecological attributes such as life history, ecological interactions, and species-specific threats. A more holistic and adaptive approach integrating regular species monitoring, habitat-level assessments, and dynamic conservation strategies is urgently needed to enhance the effectiveness of PAs for insect conservation.

The collection using Malaise traps in desert environments presented several logistical challenges that affected both deployment and data quality. Fixing traps on rocky or uneven substrates was difficult, as standard support often failed to provide stability, while in sandy areas, traps sometimes sank or shifted due to loose soil and wind, requiring frequent adjustments. Harsh weather conditions, including high temperatures and strong winds, further complicated trap maintenance and occasionally damaged nets or collection bottles. Additionally, occasional theft and limited accessibility of dune areas reduced the number of successful collections. Despite these challenges, careful placement and regular monitoring helped ensure effective sampling across the study sites.

The UAE’s network of protected areas, situated at the intersection of Afrotropical, Palearctic, and Oriental biogeographic zones, harbors distinctive insect fauna. Several newly described taxa in this study, such as *Atropatopsis abudhabiensis* (Ichneumonidae), *Ferreola alraeesii* (Pompilidae), and *Gasteruption alwathbaense* (Gasteruptiidae), display morphological traits and ecological preferences consistent with narrow endemism. The rediscovery of *Oryttus konradschmidti*, previously only known from Tunisia, further highlights biogeographic connectivity between North Africa and the Arabian Peninsula. Such discoveries emphasize the global scientific importance of the region and suggest the likely presence of additional relict or cryptic lineages. Insects, as the most diverse group of organisms on Earth, provide essential ecosystem services, including pollination, nutrient cycling, natural pest regulation, and functioning as a food base for many vertebrates. New species discoveries offer insights into evolutionary adaptations, habitat specialization, and ecological resilience in arid environments. The continued decline in insect populations globally is linked to habitat loss, climate change, and anthropogenic pressures [27]. Moreover, as [28] emphasize, insects serve as sensitive bioindicators of environmental change, and their presence or absence can signal the health of entire ecosystems.

The continued decline in insect populations globally is linked to habitat loss, climate change, and anthropogenic pressures [29]. While the UAE has undergone significant infrastructural development, its extensive protected area network, including unique wetlands, sand deserts, and mountain ecosystems, retains considerable biodiversity. However, proactive conservation efforts are now critical. These should combine species-level research with habitat protection, public education, and science-based policy frameworks to ensure the continued survival of native insect communities.

In conclusion, this study significantly advances our understanding of insect diversity in the Arabian Peninsula. It reinforces the value of long-term monitoring and habitat focused exploration in under-surveyed regions. The findings not only underscore the conservation importance of Abu Dhabi’s protected areas but also call for urgent investment in national scientific infrastructure, including the creation of a UAE Natural History Museum. Such initiatives will be vital to conserve, study, and celebrate the country’s unique biological heritage and to position the UAE as a leader in biodiversity research and conservation within the Gulf and broader Middle East.

## Figures and Tables

**Figure 1:**
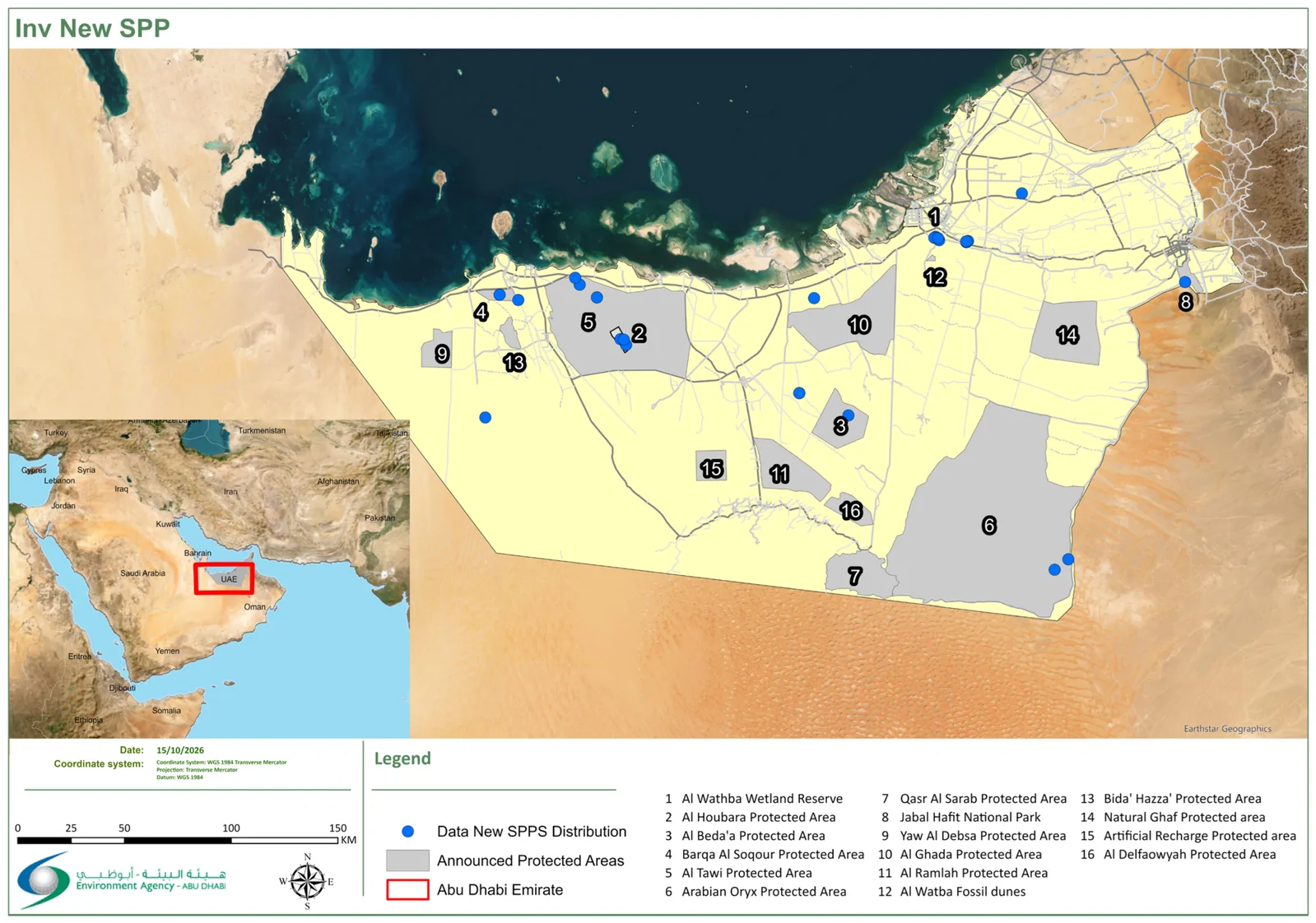
Distribution of collection localities of insect species previously described as new to science from the Abu Dhabi Emirate, United Arab Emirates.

**Figure 2:**
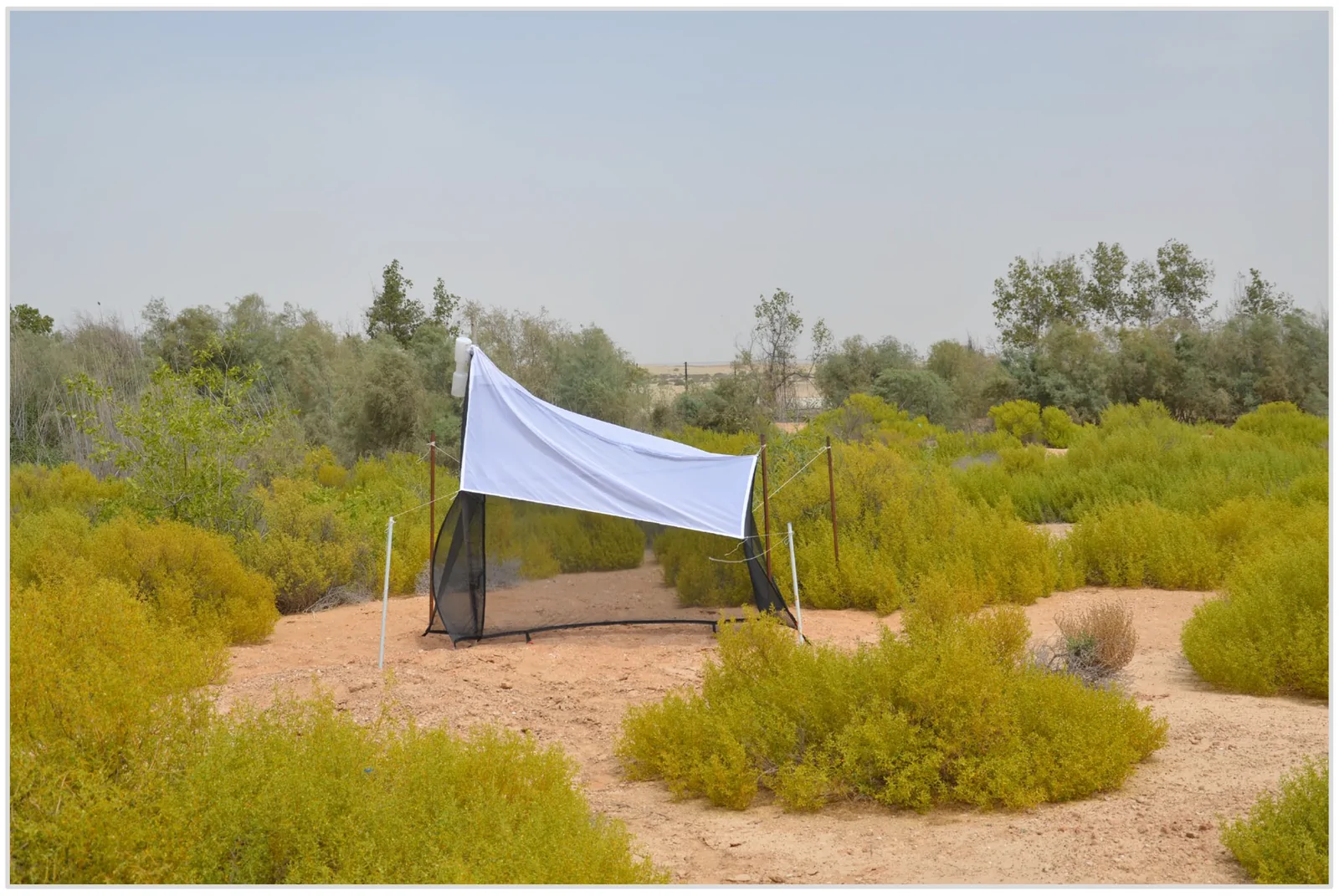
Malaise trap at AWWR (1), Emirate of Abu Dhabi, UAE, the primary collection site for most newly discovered insect species.

**Figure 3:**
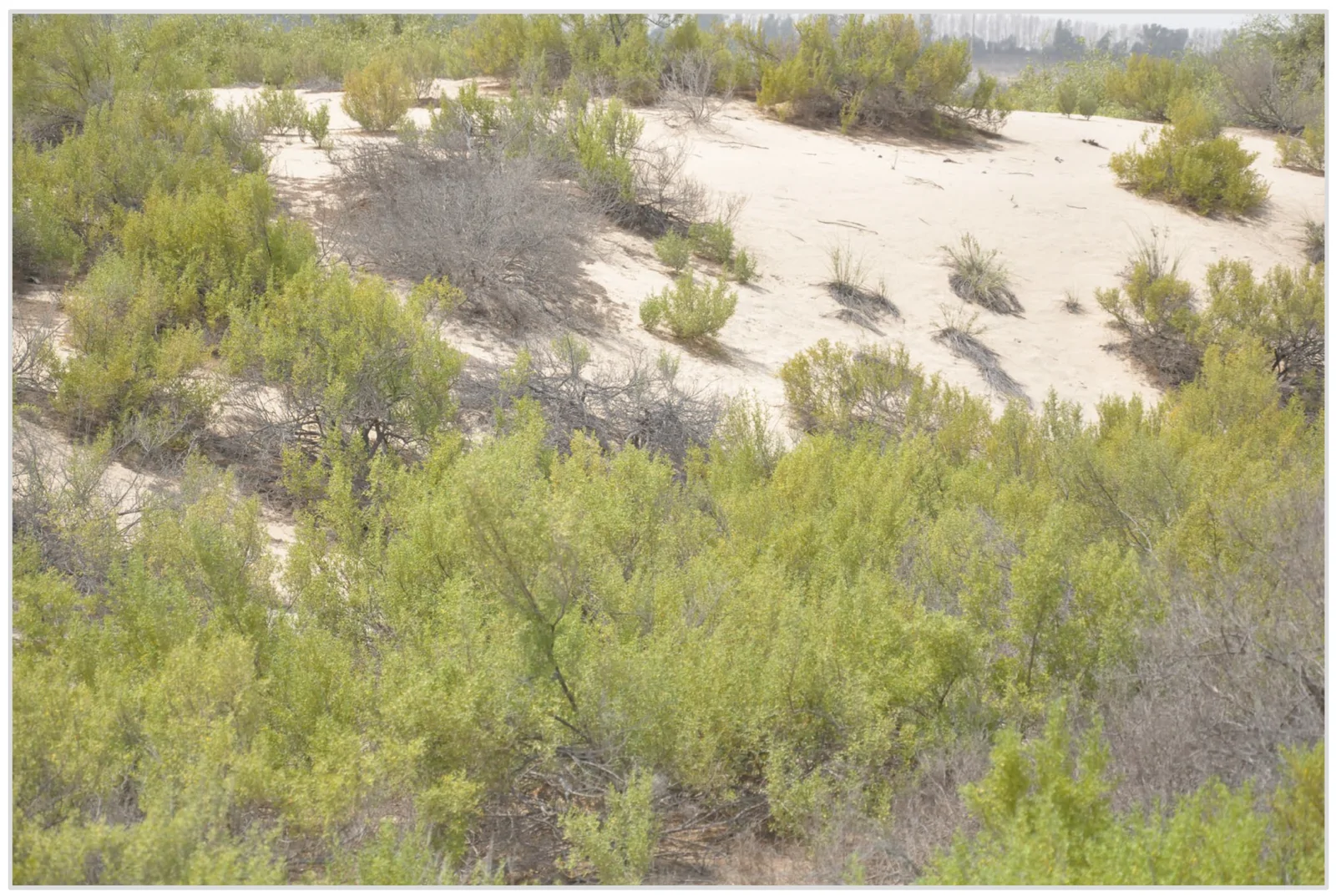
Habitat of the sampling site at AWWR (1), Emirate of Abu Dhabi, UAE.

**Figure 7:**
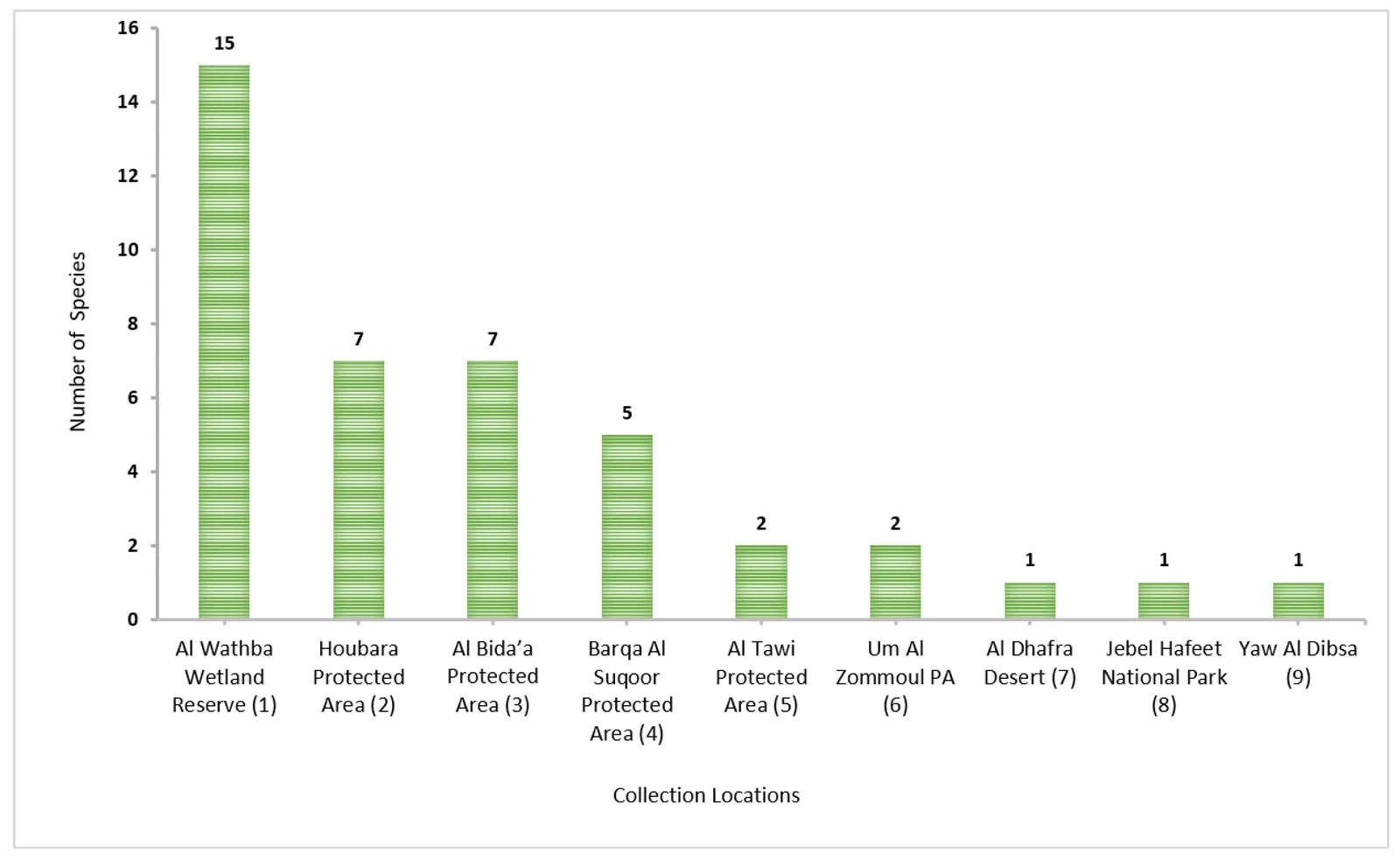
Number of species recorded from each protected area.

## Data Availability

The datasets generated and/or analyzed during the current study are available from the corresponding author upon reasonable request. All original contributions presented in this study are included in this article. Further information and inquiries can be directed to the corresponding author.

## Abbreviations

The following abbreviations are used in this manuscript:

EAD	Environment Agency–Abu Dhabi
EAD-INV	Environment Agency–Abu Dhabi (EAD) Invertebrate Collection
UAE	The United Arab Emirates
AWWR	Al Wathba Wetland Reserve
HPA	Al Houbara Protected Area
ICZN	International Code of Zoological Nomenclature
